# Progress in State Medicine

**Published:** 1903-10-24

**Authors:** 


### Progress in State Medicine.

### TUBERCULOSIS.

Pratt[1] explains that by "municipal control" of tuberculosis he means the prevention of tuberculous diseases by the action of local public authorities. The death-rate from tubercular diseases has diminished by as much as one-half during the last forty-five years, a result due to the progress in sanitary science and the efforts of local authorities. Consumption occurs in direct proportion to the amount of overcrowding and deficiency of ventilation, and prevention, therefore, turns largely upon the housing of the poor. It might be thought that overcrowding would be less in the country districts, but he found that in Leicestershire with practically the same population as the borough of Leicester, there was actually twice as much overcrowding, there being 678 tenements containing 5,761 overcrowded persons. It is important that the local authorities should recognise the importance of making consumption a notifiable disease. Good reasons exist for believing that consumption is, to a large extent, a house disease, and that the infection clings to houses, and attacks successive occupants, unless disinfection is carried out. Boards of guardians should make proper provision for the separate treatment of all consumptives. Local authorities could also do much good by providing the benefits of sanatorium treatment for early cases, while the empty wards at fever hospitals might with advantage be used for the treatment of consumptive cases. Biggs,[2] after careful estimation, places the expense of tuberculosis to the people of the United States at £66,000,000. He first calculates the loss to New York city by putting a value of £300 upon each life at the average age at which death from tuberculosis occurs. This gives a total value of the lives lost annually of £300,000. But this is not all. For at least nine months prior to death these patients cannot work, and the loss of service, together with food, nursing, medicine, attendance, etc., results in a further loss of £1,600,000, making a yearly loss to the municipality of £4,600,000. For the whole country the 150,000 deaths from tuberculosis represents in the same way £66,000,000. He states that the total expenditure in the city of New York in the care of consumptives is not at present over £100,000 a year. "If this annual expenditure were doubled or trebled, it would mean the saving of several thousand lives annually, to say nothing of the enormous saving in suffering." Further evidence of this is afforded by the fact that in the last 20 years the total number of deaths from tuberculosis in New York has decreased instead of increasing, although there has been an increase of 70 per cent. in the general population. Variot,[3] in a report to the commission on decrease of the French population, says that, "the disease which makes most victims amongst us is tuberculosis; it carries off one-third of the children. Thus in 5,500 deaths, 2,700 are due to tuberculosis in its various forms, and 1,500 to infectious diseases." The largest populations suffer most, such is the influence of contact. In 1900 the mortality in Paris increased to 5·79 per 1,000, whereas it is only 4·12 for all the united towns of France with more than 5,000 inhabitants. Numerous observations regarding the spit demonstrate that virulent germs may live for 95, 143, and 186 days. According to one of these researches the bacillus disappeared in 179 days, and to another 266. It was found at Nancy that the sun had not much influence on virulence. Guinea-pigs were infected by spit which had been 137 days in its rays, whereas similar spit, after being exposed for seven weeks to the inclemency of the weather and variations of temperature, lost all virulence. Every day guinea-pigs are injected with liquid containing dust collected by aid of sterilised linen on walls, furniture, floors of rooms inhabited by persons suffering from consumption, which frequently cause these animals to contract the disease. Street dust and that from surgeries or places into which consumptives have not entered, do not cause tuberculosis. Brousse[4] has suggested a sanitary precaution which is likely to be enforced as a general by-law for the whole of Paris. The proposal is that all laundry proprietors shall be obliged to keep special waterproof bags which shall be lent to any family having a member or friend staying in the house suffering from tuberculosis. All the linen belonging to the consumptive patients shall be placed in these bags, which must then be securely fastened. On reaching the laundries the contents must be emptied into boilers set apart for this purpose, and therein boiled with some recognised disinfectant. If this proposal cannot be adopted as a compulsory measure, it is suggested that the scheme shall be made as public as possible, and that all complying with this hygienic requirement shall be granted a subsidy. Already the Seventeenth Arrondissement of Paris has adopted it permissively. It is also suggested that medical men shall provide a simple form of certificate, mentioning that a certain patient is suffering from an infectious disease, such certificate entitling the sufferer to have his linen disinfected and washed free of cost, the laundryman using the waterproof bags and separate boiling vats, being reimbursed at a slightly higher rate by the municipality. Professor von Leyden [5] points out that sanatorium treatment is not a panacea for tuberculosis, and the work of these institutions requires to be supplemented by other means, such as dispensaries, asylums for incurable patients, and seaside homes for children. In England and France pulmonary tuberculosis has also been successfully opposed by means of general hygienic measures, but the sanatorium system is not so well developed in these countries as in Germany, where sanatoria have been established in connection with the workmen's insurance institutions. He advocates the compulsory notification of tuberculosis. At the same meeting Herr Pütter, Town Councillor of Halle, described the work of the Anti-tuberculosis Association there. Once a week the medical officer examined patients sent to him by the president of the association. Thermometers were given to them and they were shown how to take their temperature, those who could not do this properly being visited at their homes by nurses and deaconesses connected with the association. The sputa were examined at the municipal laboratory free of charge. The patients also received printed instructions on the subject of hygiene, and in certain cases money to be spent on food ; they were also supplied with various articles of table service, so that they need not have recourse to those which were used by the other members of the family. In the case of unmarried persons living alone, the association took lodgings for them and disinfected the rooms with formalin from time to time. The association worked in harmony with the University clinics and other hospitals, and also with the medical men who were quite willing to hand over the care of their poorer patients.

[1] Sanitary Rec., March 19, 1903. [2] Public Health, April 1903 [3] Public Health Engineer, April 25, 1903. [4] Sanitary Rec., April 16, 1903. [5] Lancet, May 30, 1903.

*(To be concluded.)*

